# Comparing single- and multi-post labeling delays for the measurements of resting cerebral and hippocampal blood flow for cerebrovascular testing in midlife adults

**DOI:** 10.3389/fphys.2024.1437973

**Published:** 2024-10-02

**Authors:** Kevin P. Decker, Faria Sanjana, Nick Rizzi, Mary K. Kramer, Alexander M. Cerjanic, Curtis L. Johnson, Christopher R. Martens

**Affiliations:** ^1^ Department of Kinesiology and Applied Physiology, University of Delaware, Newark, DE, United States; ^2^ Department of Biomedical Engineering, University of Delaware, Newark, DE, United States; ^3^ Department of Neurology, Massachusetts General Hospital, Boston, MA, United States

**Keywords:** brain health, cerebral blood flow (CBF), cerebrovascular reactivity (CVR), magnetic resonance imaging (MRI), and arterial spin labeling (ASL) MRI

## Abstract

**Objectives:**

To assess the reliability and validity of measuring resting cerebral blood flow (CBF) and hippocampal CBF using a single-post-labeling delay (PLD) and a multi-PLD pseudo-continuous arterial spin labeling (pCASL) protocol for cerebrovascular reactivity (CVR) testing.

**Methods:**

25 healthy, midlife adults (57 ± 4 years old) were imaged in a Siemens Prisma 3T magnetic resonance imaging (MRI) scanner. Resting CBF and hippocampal CBF were assessed using two pCASL protocols, our modified single-PLD protocol (pCASL-MOD) to accommodate the needs for CVR testing and the multi-PLD Human Connectome Project (HCP) Lifespan protocol to serve as the reference control (pCASL-HCP). During pCASL-MOD, CVR was calculated as the change in CBF from rest to hypercapnia (+9 mmHg increase in end-tidal partial pressure of carbon dioxide [P_ET_CO_2_]) and then normalized for P_ET_CO_2_. The reliability and validity in resting gray matter (GM) CBF, white matter (WM) CBF, and hippocampal CBF between pCASL-MOD and pCASL-HCP protocols were examined using correlation analyses, paired t-tests, and Bland Altman plots.

**Results:**

The pCASL-MOD and pCASL-HCP protocols were significantly correlated for resting GM CBF [r = 0.72; F (1, 23) = 25.24, *p* < 0.0001], WM CBF [r = 0.57; F (1, 23) = 10.83, *p* = 0.003], and hippocampal CBF [r = 0.77; F (1, 23) = 32.65, *p* < 0.0001]. However, pCASL-MOD underestimated resting GM CBF (pCASL-MOD: 53.7 ± 11.1 v. pCASL-HCP: 69.1 ± 13.1 mL/100 g/min; *p* < 0.0001), WM CBF (pCASL-MOD: 32.4 ± 4.8 v. pCASL-HCP: 35.5 ± 6.9 mL/100 g/min; *p* = 0.01), and hippocampal CBF (pCASL-MOD: 50.5 ± 9.0 v. pCASL-HCP: 68.1 ± 12.5 mL/100 g^/^min; *p* < 0.0001). P_ET_CO_2_ increased by 8.0 ± 0.7 mmHg to induce CVR (GM CBF: 4.8% ± 2.6%; WM CBF 2.9% ± 2.5%; and hippocampal CBF: 3.4% ± 3.8%).

**Conclusion:**

Our single-PLD pCASL-MOD protocol reliably measured CBF and hippocampal CBF at rest given the significant correlation with the multi-PLD pCASL-HCP protocol. Despite the lower magnitude relative to pCASL-HCP, we recommend using our pCASL-MOD protocol for CVR testing in which an exact estimate of CBF is not required such as the assessment of relative change in CBF to hypercapnia.

## Introduction

The human brain receives 15%–20% of total cardiac output to meet its metabolic demands. Resting cerebral blood flow (CBF) can be a prognostic and diagnostic factor in determining brain health (Scheef et al., 2010; Tosun et al., 2010; Wierenga et al., 2014) as alterations or abnormalities in resting CBF are associated with mild cognitive impairment and can precede clinical dementia (Alexopoulos et al., 2012; Wolters et al., 2017). Disruptions in resting CBF contribute to the pathophysiology of Alzheimer’s disease (AD) and related dementias, even in the absence of amyloid-β accumulation or brain atrophy (Iadecola, 2004; Ruitenberg et al., 2005). When matched for age, AD patients demonstrate a ∼40% decrease in resting CBF compared to cognitively normal adults (Asllani et al., 2008).

Among the techniques to measure CBF, magnetic resonance imaging (MRI) offers superior spatial resolution whereas other techniques (e.g., transcranial Doppler, near-infrared spectroscopy) have better temporal resolution but are limited to estimations from a single cerebral artery or brain region. MRI can also distinguish between perfusion of gray matter (GM), the brain tissue most involved in information processing because it contains a high density of neuronal cell bodies and dendrites, and white matter (WM), the myelinated neuronal tracts used for communication between gray matter regions (Filley, 2012). An important region of interest concerning AD risk includes the hippocampus, a brain structure responsible for memory encoding and recall that is affected by normal aging, cognitive impairment, and AD (Fjell et al., 2014).

Arterial spin labeling (ASL) is an MRI technique that non-invasively (i.e., without an exogenous contrast agent) labels water in arterial blood using magnetic radiofrequency pulses to measure cerebral perfusion (Alsop et al., 2010; Alsop et al., 2015; Melzer et al., 2011). The basic concept of ASL is to use pairwise subtraction of control images (noninverted blood proton magnetization) and labeled images (inverted blood proton magnetization) to provide a signal intensity that is converted to CBF (Haller et al., 2016). Pseudo-continuous ASL (pCASL) is a variation of ASL that administers a train of short radiofrequency pulses to emulate continuous labeling and thus improve the signal-to-noise ratio and increase reproducibility (Alsop et al., 2015; Wu et al., 2007; Dai et al., 2008; Chen et al., 2011). Collectively, ASL and pCASL are validated and well-correlated with the gold-standard H_2_^15^O positron emission tomography scan to measure CBF (Donahue et al., 2006; Xu et al., 2010; Kamano et al., 2013; Heijtel et al., 2014).

In addition to CBF at rest being a measurement of brain vascular health, cerebrovascular reactivity (CVR) offers a more dynamic assessment. CVR quantifies the increase in CBF from rest in response to a vasodilatory challenge, which reflects the ability of cerebral blood vessels to respond to altered homeostasis. (Fierstra et al., 2013; Fisher and Mikulis, 2021). CVR has the potential to be more sensitive than CBF alone in predicting cognitive performance in adults without cognitive impairment (Kim et al., 2021). One type of vasodilatory challenge is the delivery of air mixed with CO_2_ to induce hypercapnia (i.e., increased PaCO_2_) which promotes the increase in CBF. Hypercapnia is well-tolerated for short periods and is typically applied for less than 5 min to avoid uncomfortable side effects from prolonged exposure such as headache, dizziness, and hyperventilation.

A current dilemma for researchers seeking to quantify CBF is that many different imaging protocols exist, thus making the relatability between studies low. In an attempt to harmonize imaging protocols across sites, a series of guidelines on imaging and analysis protocols have been published by the Human Connectome Project (HCP). The HCP is a major, NIH-funded, multi-site neuroimaging initiative designed to acquire multimodal imaging data related to the brain’s structural and functional connectivity that could be adopted by other neuroimaging laboratories as a reference standard for acquiring high-quality, high-resolution data (Van Essen et al., 2012; Van Essen et al., 2013; Uğurbil et al., 2013; Glasser et al., 2016). More recently, the HCP has expanded their guidelines to characterize CBF using a multi-post labeling delay (PLD) pCASL protocol implemented with a 2D simultaneous multishot echo planar imaging (SMS-EPI) pCASL pulse sequence across the lifespan (Li et al., 2015; Harms et al., 2018; Somerville et al., 2018; Bookheimer et al., 2019; Juttukonda et al., 2021). The multi-PLD used in the pCASL-HCP improves the accuracy of the CBF measurement by measuring the arterial transit time (ATT) or the time for the labeled blood to reach the brain tissue being imaged.

While pCASL-HCP is regarded as a benchmark protocol for measuring resting CBF, the longer scan time of this protocol required by the multi-PLD is less desirable for protocols involving CVR, which require fine-tuning of scan timings to fit the duration of the hypercapnia challenge. To address this issue, our laboratory implemented a single-PLD protocol using the recommendations of the International Society on Magnetic Resonance in Medicine (ISMRM) white paper (Alsop et al., 2015) to examine CVR (pCASL-MOD). Single-PLD protocols are often used in clinical practice due to time, availability, and simplicity in the measurement (Clement et al., 2022). Indeed, a major goal of pCASL-MOD with one PLD is to achieve the temporal resolution necessary to examine CBF kinetics throughout the CVR test (e.g., minute-by-minute). This increase in temporal resolution comes at the expense of the limited ability to account for variations in ATT across brain regions due to vascular anatomy.

The purpose of this study was to determine the reliability and validity of resting CBF and hippocampal CBF between the pCASL-MOD protocol with a single PLD used during CVR testing and the pCASL-HCP protocol with multiple PLDs. We hypothesized that our pCASL-MOD protocol would be well-correlated with pCASL-HCP in measuring CBF and hippocampal CBF at rest but may underestimate the absolute value of pCASL-HCP due to the single-PLD not being able to capture peak blood flow to regions with ATT that varied from the global average. Furthermore, we describe the changes in CBF seen in the GM, WM, and hippocampus during CVR testing and recommendations on using our single-PLD protocol (pCASL-MOD).

## Methods

### Study participants

Twenty-five (25) healthy midlife adults, defined as the age of 50–64 years old (10 males/15 females) were recruited for this study from Newark, Delaware, and surrounding areas. All participants were non-smokers and free from chronic diseases and major psychological or neurological disorders as assessed by medical history questionnaires. None consumed medications or supplements known to lower blood pressure, triglycerides, or cholesterol. Participants were excluded for obesity defined as body mass index (BMI) > 30 kg/m^2^ and body fat >25% for men or >33% for women or if they had stage I hypertension or higher, defined as systolic blood pressure >130 mmHg or diastolic blood pressure >90 mmHg. Participants were also excluded if unable or unwilling to participate in an MRI scan (e.g., metal implants, claustrophobia). Women were at least 1 year post menopause and not receiving any hormone replacement therapy. Informed consent was obtained from all participants. The study was approved by the University of Delaware’s Institutional Review Board and all experimental testing conformed to the standards outlined in the Declaration of Helsinki.

### Testing session

**FIGURE 1:**
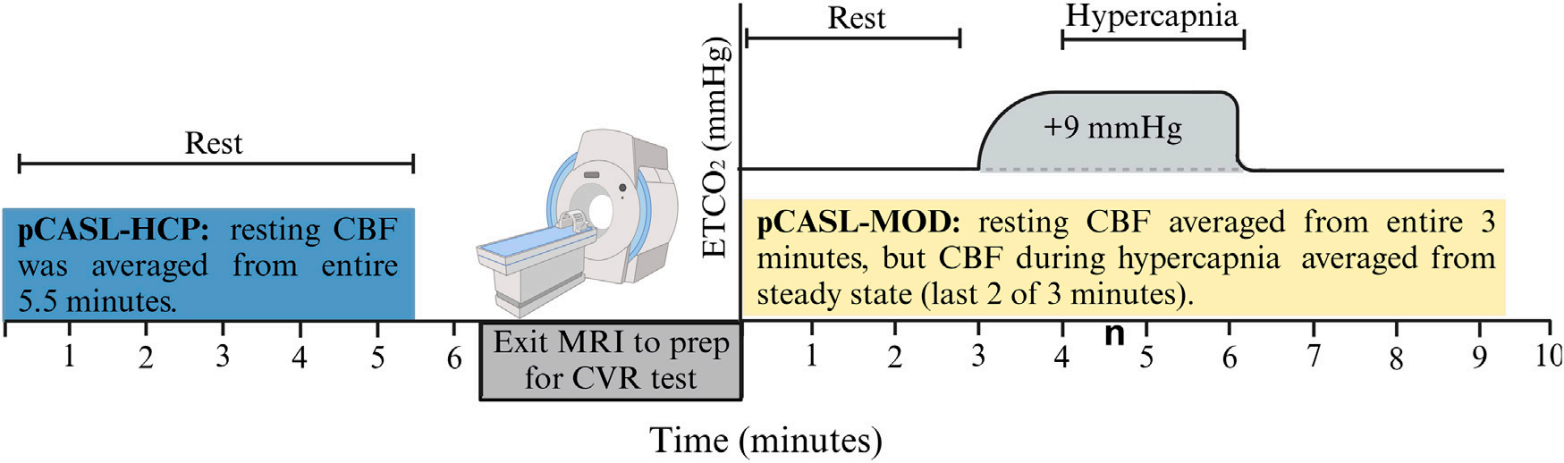
Schematic of the testing session. Resting cerebral blood flow (CBF) was measured with the pCASL-HCP protocol, and participants exited MRI to prep for cerebrovascular reactivity (CVR) test with the pCASL-MOD protocol.

Participants reported to the laboratory in the morning (07:00–09:00) after an overnight fast (>8 h) and refrained from alcohol and vigorous exercise for 24 h and over-the-counter medications for 48 h. Height, weight, and blood biomarkers were measured before the MRI brain scan, as seen in Figure 1. Blood was sampled from the antecubital vein and sent to a commercial clinical laboratory (LabCorp) for reporting of basic subject characteristics.

### MRI acquisition

Brain scans used a 3T Siemens Prisma MRI scanner with a 64-channel head coil (Siemens, Erlangen, Germany). Participants were in the supine position with a foam pad under the legs for comfort and instructed not to move throughout the scan. High-spatial-resolution volumetric T1-weighted magnetization-prepared rapid acquisition gradient-echo (MPRAGE) anatomical images of the brain were acquired with the following parameters: repetition time (TR)/inversion time (TI)/echo time (TE) = 2,300/900/2.32 ms, spatial resolution = 0.9 × 0.9 × 0.9 mm (Wierenga et al., 2014) in-plane spatial resolution, 8° flip angle, 192 slices per slab.

#### CBF from pCASL-HCP protocol

The pCASL-HCP is the multi-PLD protocol that measured resting CBF, as previously described (Li et al., 2015; Harms et al., 2018; Bookheimer et al., 2019; Juttukonda et al., 2021). Imaging parameters used for the pCASL-HCP protocol were as follows: TR = 3,705 ms; TE = 26.4 ms; voxel size = 2.5 × 2.5 × 2.3 mm^3^; 90° flip angle, 215 × 215 mm^2^ FOV; 86 x 86 matrix, 43 slices, slice thickness 2.27 mm; 43 pairs of control/label images; total acquisition time = 5.5 min. The labeling duration was 1,500 ms with five PLDs = 200 ms (control/label pairs = 6), 700 ms (pairs = 6), 1,200 ms (pairs = 6), 1,700 ms (pairs = 10), and 2,200 ms (pairs = 15). Signal readout was implemented using 2D multi-band gradient-echo EPI using partial Fourier = 6/8 and SMS acceleration factor = 1.

#### CBF from pCASL-MOD

The pCASL-MOD is a single-PLD protocol, modified to agree with the ISMRM white paper (Alsop et al., 2015) used for measuring CBF at rest and during the CVR testing. Imaging parameters used for pCASL-MOD protocol were as follows: TR = 4,510 ms; TE = 20 ms; voxel size: 2.5 × 2.5 × 2.3 mm^3^; flip angle: 90°; 215 × 215 mm^2^ FOV; 86 x 86 matrix; 60 slices; slice thickness: 2.27 mm; 60 pairs of control/label images; total acquisition time = 9.5 min. A labeling duration of 1,800 ms with a single-PLD of 1,800 ms. The signal readout was implemented using 2D multi-band gradient-echo EPI using partial Fourier = 6/8 and SMS acceleration factor = 6.

The pCASL-MOD was designed to measure CBF during the CVR test consisting of 3 min of simulated room air (rest), 3 min of air mixed with +9 mmHg increase in end-tidal partial pressure of carbon dioxide [P_ET_CO_2_] (hypercapnia), and 3 min of simulated room air (recovery). Hypercapnia was achieved using an MR-compatible prospective end-tidal targeting system which allows for precise and repeatable manipulation of P_ET_CO_2_ on a breath-by-breath basis using a computerized gas blender (RespirAct™) (Mark et al., 2010; Winter et al., 2010; Fisher, 2016). The soft plastic mask was sealed to the face using an adhesive tape designed for use on the skin (Tegaderm 3M Healthcare, St. Paul, MN, United States), with an additional 6-inch tubing fed through the head coil to connect with the tubing circuit of the RespirAct™ unit. Participants were asked to breathe deeply and consistently to accurately and continuously target PETCO2. To measure minute-by-minute CBF during the 9-min CVR test, 6 to 7 control/label pairs from pCASL-MOD were analyzed per minute. Resting CBF was averaged over the entire 3 min (20 control/label pairs) and CBF during hypercapnia was averaged from the last 2 of 3 min to reach a steady state (12–14 control/label pairs). CVR was calculated as the percent change in resting CBF to hypercapnia The percent increase in hypercapnia-induced CBF was then normalized for P_ET_CO_2_ change in mmHg as follows: CVR = 100 × [((CBF_Hypercapnia_ – CBF_rest_)/CBF_rest_)/ΔP_ET_CO_2_].

### Image processing

CBF maps were obtained with a standard kinetic-model inversion using the Bayesian algorithm under the graphic user interface BASIL in FSL (Chappell et al., 2009). For both the pCASL-MOD and pCASL-HCP protocols, two equilibrium echo magnetization (M0) images were acquired at the scan’s end and averaged to produce a calibration image. Perfusion maps were calibrated onto the proton density-weighted M0 image using voxel-wise calibration mode with a sequence TR = 8 s and a calibration gain = 1. CBF for each voxel was calculated using the following formula:

$$CBF=\frac{6000*\;\lambda \;*\left({SI}_{control}-\;{SI}_{label}\right)*\;e^{\frac{PLD}{T_{1b}}}}{2*\;\alpha *\;T_{1b}*\;{SI}_{PD}*\left(1-\;e^{-\frac{\tau }{T_{1b}}}\right)}\;\left[ml/100g/\min\right]$$

where λ = 0.9 mL/g (brain/blood barrier partition coefficient); SI_control_ and SI_label_ = time-averaged signal intensities of control and label images; T_1b_ = 1.65 s (longitudinal relaxation time of blood); α = 0.85 (labeling efficiency); SI_PD_ = signal intensity of proton density-weighted image; τ = labeling duration. The factor of 6,000 was used to convert the units from mL/g/s to mL/100 g/min (Alsop et al., 2015).

### Statistical analysis

Participant anthropometric and clinical laboratory values are presented as the mean ± standard deviation. Shapiro–Wilk tests were used to evaluate the normality of GM CBF, WM CBF, hippocampal CBF, and P_ET_CO_2_ responses. A Pearson correlation coefficient assessed the strength of our single-PLD protocol (pCASL-MOD) compared to the reference multi-PLD protocol (pCASL-HCP). Paired sample t-tests and Bland Altman analysis (95% limits of agreement) were used to compare differences between pCASL-MOD and pCASL-HCP. Further, paired sample t-tests were used to compare differences in P_ET_CO_2_ and CBF from rest to hypercapnia during CVR testing. Statistical analysis and figures were generated using GraphPad Prism 8.0 (GraphPad Software, San Diego, California United States) with statistical significance determined at *p* < 0.05.

## Results

**FIGURE 2:**
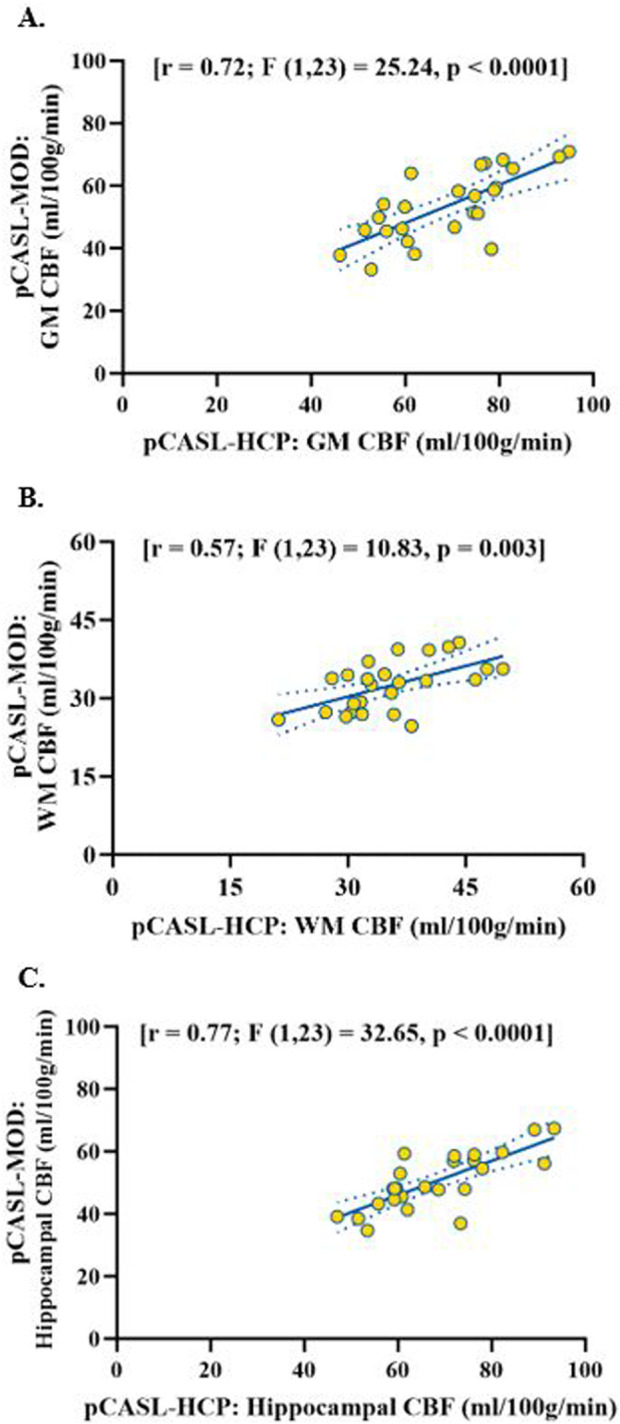
Correlation of resting cerebral blood flow between pCASL-MOD and pCASL-HCP in gray matter **(A)**, white matter **(B)** and hippocampal **(C)**. N = 25 participants.

**FIGURE 3:**
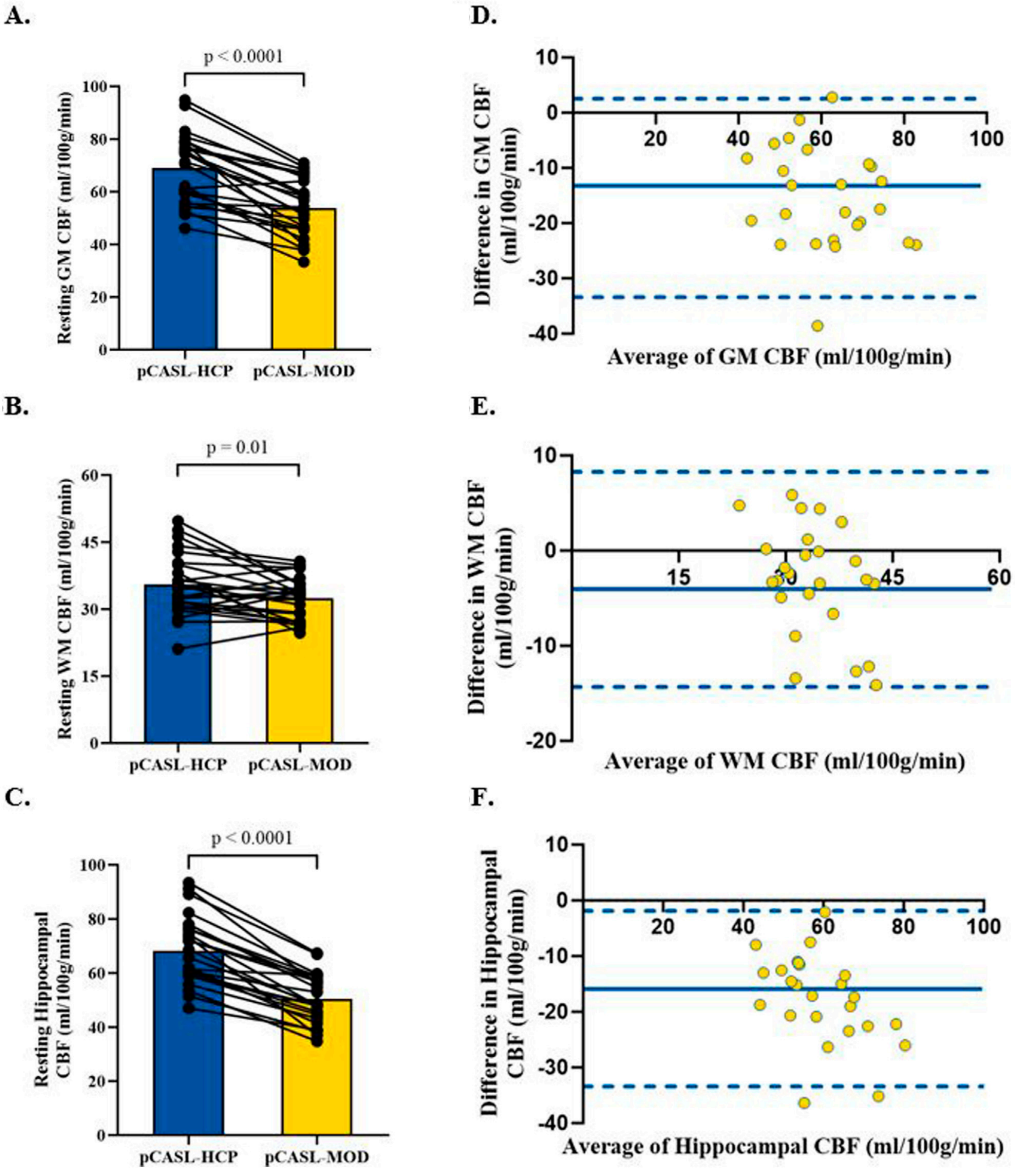
Comparison in resting cerebral blood flow between pCASL-MOD and pCASL-HCP in gray matter **(A)**, white matter **(B)**, and hippocampal **(C)**. Bland Altman plots of resting cerebral blood flow between pCASL-MOD and pCASL-HCP showing a mean difference in gray matter **(D),** white matter **(E),** and hippocampal **(F).** N = 25 participants. Data presented as mean ± standard deviation.

**FIGURE 4:**
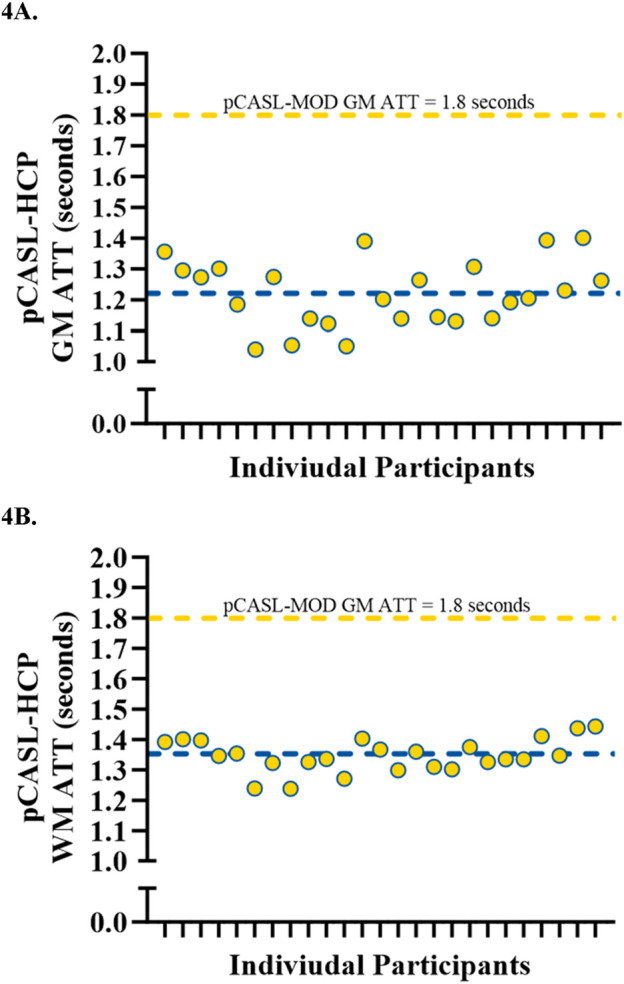
Individual data showcasing the variations in arterial transit time measured from pCASL-HCP being compared to fixed pCASL-MOD in gray matter **(A)** and white matter **(B)**. N = 25 participants.

Participant characteristics are presented in Table 1. The pCASL-MOD protocol was significantly correlated with pCASL-HCP in resting GM CBF (Figure 2A; r = 0.72; F (1, 23) = 25.24, *p* < 0.0001), WM CBF (Figure 2B; r = 0.57; F (1, 23) = 10.83, *p* = 0.003), and hippocampal CBF (Figure 2C; r = 0.77; F (1, 23) = 32.65, *p* < 0.0001). Compared to pCASL-HCP, pCASL-MOD underestimated GM CBF (Figure 3A; pCASL-MOD: 53.7 ± 11.1 v. pCASL-HCP: 69.1 ± 13.1 mL/100 g/min; *p* < 0.0001), WM CBF (Figure 3B; pCASL-MOD: 32.4 ± 4.8 v. pCASL-HCP: 35.5 ± 6.9 mL/100 g/min; *p* = 0.01), and hippocampal CBF (Figure 3C; pCASL-MOD: 50.5 ± 9.0 v. pCASL-HCP: 68.1 ± 12.5 mL/100 g^/^min; *p* < 0.0001). The Bland Altman plots report a mean difference in GM CBF (Figure 3D; −15.4 ± 9.2 mL/100 g^/^min), WM CBF (Figure 3E; −3.0 ± 5.8 mL/100 g^/^min), and hippocampal CBF (Figure 3F; −17.6 ± 8.1 mL/100 g^/^min) between pCASL-MOD and pCASL-HCP protocols. While ATT from single-PLD pCASL-MOD protocol was fixed at 1.8 s, the multi-PLD pCASL-HCP protocol measures GM ATT (Figure 4A; 1.22 ± 0.11 s) and WM ATT (Figure 4B; 1.35 ± 0.05 s).

**TABLE 1:** Subject characteristics.

Variable	Mean ± standard deviation
Sex (male/female)	10/15
Age (years)	57 ± 4
Body Mass (kg)	72.3 ± 14.2
Height (cm)	170.0 ± 8.5
Body Mass Index (kg/m^2^)	25.1 ± 3.6
Systolic Blood Pressure (mmHg)	114 ± 9
Diastolic Blood Pressure (mmHg)	69 ± 7
Mean Arterial Blood Pressure (mmHg)	84 ± 7
Heart Rate (beats per minute)	64 ± 7
Triglycerides (mg/dL)	91 ± 24
Total Cholesterol (mg/dL)	203 ± 26
LDL Cholesterol (mg/dL)	122 ± 22
HDL Cholesterol (mg/dL)	61 ± 19

**FIGURE 5:**
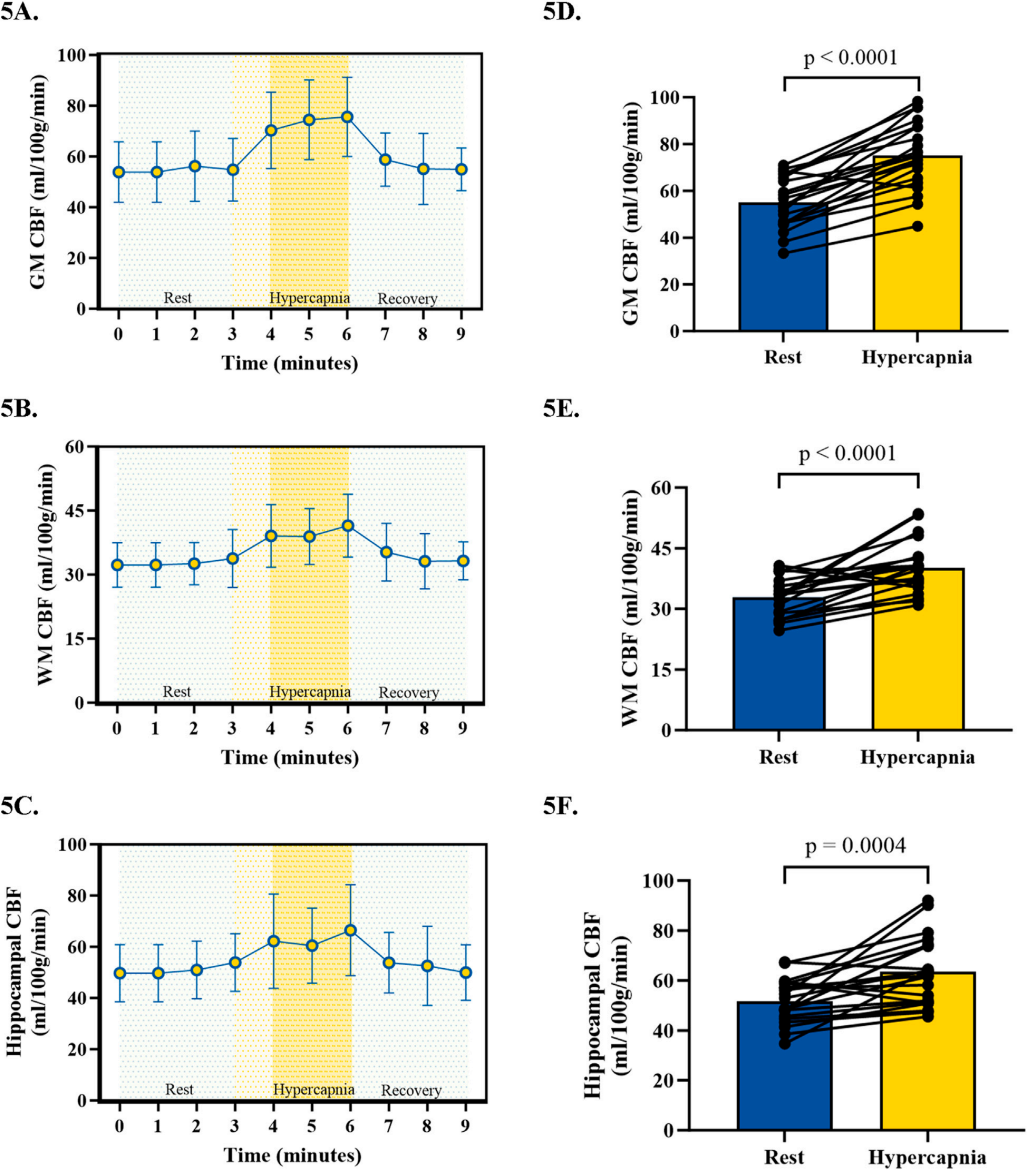
Kinetics of cerebral blood flow during cerebrovascular reactivity testing for gray matter **(A)**, white matter **(B)**, and hippocampal **(C)**. The hyperemic response in cerebral blood flow from 3 min of rest to the last 2 min of hypercapnia for gray matter **(D)**, white matter **(E)**, and hippocampal **(F)**. N = 21 participants. Data presented as mean ± standard deviation.

**FIGURE 6:**
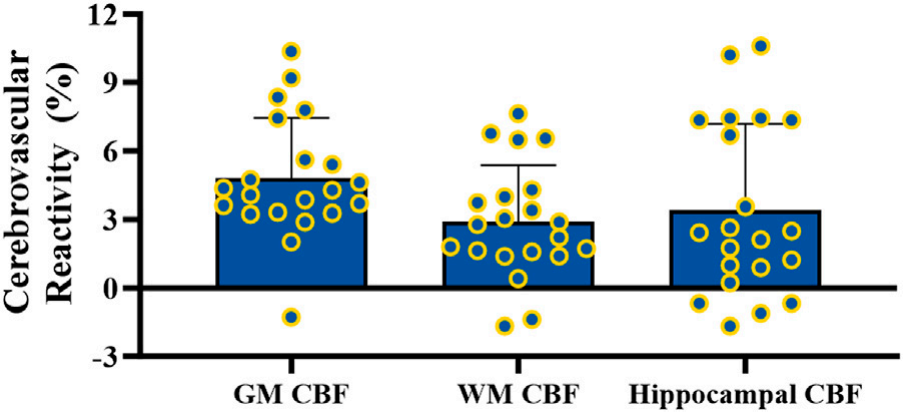
Cerebrovascular reactivity testing for gray matter, white matter**,** and hippocampal cerebral blood flow. N = 21 participants. Data presented as mean ± standard deviation.

During CVR testing with our pCASL-MOD protocol, four participants were excluded from analysis due to an inability to achieve our laboratory’s predefined cutoff for a sufficient increase in P_ET_CO_2_ (≥6.5 mmHg). In the 21 participants, the GM CBF, WM CBF, and hippocampal CBF kinetics during the CVR test on a minute-by-minute basis were reported in Figures 5A–C, respectively. P_ET_CO_2_ increased by 8.0 ± 0.7 mmHg (rest: 38.8 ± 3.72 hypercapnia: 46.7 ± 3.4 mmHg; *p* < 0.001) which increased GM CBF by 20.1 ± 11.0 mL/100 g/min (Figure 5D; rest: 55.0 ± 11.0; hypercapnia: 75.1 ± 14.4 mL/100 g^/^min; *p* < 0.0001), WM CBF by 7.3 ± 6.7 mL/100 g/min (Figure 5E; rest: 32.9 ± 4.8; hypercapnia: 40.2 ± 6.4 mL/100 g^/^min; *p* < 0.0001), and hippocampal CBF by 11.8 ± 13.3 mL/100 g/min (Figure 5F; rest: 51.7 ± 8.7; hypercapnia: 63.5 ± 13.5 mL/100 g^/^min; *p* = 0.0004). CVR values are reported after being normalized for P_ET_CO_2_ (Figure 6; GM CBF: 4.8% ± 2.6%; WM CBF 2.9% ± 2.5%; and hippocampal CBF: 3.4% ± 3.8%). A summary of P_ET_CO_2_ and CBF responses from CVR testing during pCASL-MOD can be found in Table 2.

**TABLE 2:** End-tidal CO_2_ and cerebral blood flow responses to air mixed with CO_2_ to induce hypercapnia for cerebrovascular reactivity testing. N = 21 participants. Data presented as mean ± standard deviation.

Variable	Mean ± standard deviation
Resting end-tidal CO_2_ (mmHg)	38.8 ± 3.2
Hypercapnia end-tidal CO_2_ (mmHg)	46.7 ± 3.4
Change in end-tidal CO_2_ (mmHg)	8.0 ± 0.7
Resting GM CBF (ml/100 g/min)	55.0 ± 11.0
Hypercapnia GM CBF (ml/100 g/min)	75.0 ± 14.4
Change in GM CBF (ml/100 g/min)	20.1 ± 10.8
GM CBF cerebrovascular reactivity (%)	4.8 ± 2.6
Resting WM CBF (ml/100 g/min)	32.9 ± 4.8
Hypercapnia WM CBF (ml/100 g/min)	40.2 ± 6.4
Change in WM CBF (ml/100 g/min)	7.3 ± 6.7
WM CBF cerebrovascular reactivity (%)	2.9 ± 2.5
Resting hippocampal CBF (ml/100 g/min)	51.7 ± 8.7
Hypercapnia hippocampal CBF (ml/100 g/min)	63.5 ± 13.5
Change in hippocampal CBF (ml/100 g/min)	11.8 ± 13.2
Hippocampal CBF cerebrovascular reactivity (%)	3.1 ± 3.4

### Discussion

Resting cerebral blood flow (CBF) and cerebrovascular reactivity (CVR) are important physiological parameters for determining brain health and risk for cognitive impairment and dementia. The purpose of this study was to assess the reliability and validity of resting CBF and hippocampal CBF in healthy midlife adults using a modified single-post-labeling delay (PLD) protocol (pCASL-MOD) that we specifically designed for CVR testing. To do so, we compared our pCASL-MOD with the standard multi-PLD protocol from the Human Connectome Project (pCASL-HCP). The main finding from this study is that resting gray matter (GM) CBF, white matter (WM) CBF, and hippocampal CBF from our pCASL-MOD are significantly correlated with measures from pCASL-HCP. The finding that the pCASL-MOD protocol underestimated CBF compared to the pCASL-HCP protocol agrees with our hypothesis. Therefore, we can only safely recommend the use of our pCASL-MOD protocol for CVR testing in which an exact estimate of CBF is not required such as the assessment of relative change in CBF to hypercapnia.

#### Resting cerebral blood flow

This study utilized a within-subjects design to measure GM CBF, WM CBF, and hippocampal CBF using two distinct arterial spin labeling (ASL) protocols, employing single vs. multiple PLDs. The pCASL-MOD was intended specifically for CVR testing, and unlike pCASL-HCP, required different parameters used according to the recommendations of the International Society on Magnetic Resonance in Medicine (ISMRM) white paper (Alsop et al., 2015). However, we sought to determine whether resting CBF was comparable to the established pCASL-HCP protocol (Harms et al., 2018; Somerville et al., 2018; Bookheimer et al., 2019; Juttukonda et al., 2021). Our CBF data obtained using the pCASL-HCP protocol was slightly higher than previous reports from a similar age-matched group (Juttukonda et al., 2021). Variability is common in reports of CBF using MRI, even in age-matched studies (Calamante et al., 1999), an effect that can be derived from small sample sizes and perfusion-modifying factors such as biological sex, and blood composition and components (Clement et al., 2018). To limit the variability in CBF using different imaging protocols, the Human Connectome Project was developed to provide precision and uniformity in measuring resting CBF using a multi-PLD protocol. However, this multi-PLD requires longer scan times and does not provide the temporal resolution to examine CBF during CVR testing.

The primary difference between our modified protocol and that of the Human Connectome Project is that ours utilizes a single-PLD whereas the pCASL-HCP uses multiple PLDs. The PLD is the time delay between the end of the labeling pulse and the start of image acquisition, which can affect measurement accuracy as there is variability between individuals in how long it takes blood to flow from the labeling plane to the imaging plane. The observed discordance in resting CBF between the two protocols is most likely explained by the differences in PLD between the two protocols. Quantification of CBF from a single-PLD protocol (pCASL-MOD) uses a model that assumes the arterial transit time (ATT) to be shorter than the PLD. Single-PLD protocols are more often recommended in the clinical setting due to being short, direct, and reliable (Alsop et al., 2015). In contrast, a multi-PLD protocol can account for ATT heterogeneity between participants and specific regions of the brain, using the measured ATT to inform a more accurate two-compartment model which provides more precise quantification of CBF (Wu et al., 2014). Therefore a multi-PLD protocol, or more specifically the pCASL-HCP, was elected as our reference control protocol (Alsop et al., 2015; Mezue et al., 2014). Our data agrees with simulated data that a single-PLD protocol will underestimate CBF compared to a multi-PLD protocol (Bladt et al., 2020). Our work focuses on midlife adults due to their susceptibility to future cerebrovascular and cognitive risk factors. Therefore, the effect of aging on the reduction in blood velocity and longer ATTs in the carotid arteries (Kochanowicz et al., 2009) could have further implications on the PLD and accuracy of CBF compared to younger individuals (Hu et al., 2020). In all of our midlife participants, ATT measured from pCASL-HCP in GM and WM was less than the fixed ATT from our pCASL-MOD, which could contribute to the discrepancy in CBF values.

Despite the underestimation of resting CBF, our pCASL-MOD protocol was significantly correlated with the pCASL-HCP protocol for GM CBF, WM CBF, and hippocampal CBF. While CBF is an important clinical marker, it lacks the dynamic and integrative information that can be gathered by observing the cerebral blood vessels in response to stimuli during CVR. Previous work has noted the association between CVR impairment and increased risk of death (Portegies et al., 2014). While the pCASL-HCP protocol is the preferred method for measuring CBF at rest, we do not suggest our pCASL-MOD protocol to serve as a replacement. Instead, we recommend the use of our pCASL-MOD protocol for CVR testing in which an exact estimate of CBF is not required such as the assessment of relative change in CBF to hypercapnia.

#### Cerebrovascular reactivity

The inclusion of CVR testing in cerebrovascular research holds promising value as it is more sensitive than resting CBF alone for predicting cognitive performance in adults at risk of cognitive decline (Kim et al., 2021). To date, only a few studies have investigated hypercapnia-induced CVR using pCASL techniques (De Vis et al., 2015; Zhou et al., 2015). A major advantage of our CVR protocol includes the use of a system to control CO_2_ delivery and improved spatiotemporal imaging resolution. Using a prospective end-tidal targeting system to control CO_2_, we can use MRI to obtain the temporal (e.g., minute-to-minute) and spatial (e.g., regions of interest such as the hippocampus) resolution to measure changes in CBF during hypercapnia. While the current study provides novelty in reporting GM and WM CBF, our laboratory is interested in researching hippocampal CBF. The hippocampus has a unique role in memory encoding and recall (Fjell et al., 2014), making hippocampal CBF and its responses to CVR insightful for cerebrovascular research to understand the pathophysiology of AD and related dementias.

While many stimuli can provoke an increase in CBF, administration of hypercapnia is the preferred method for CVR testing because it is non-invasive, uses a potent and rapid vasodilatory stimulus, and quickly returns to baseline conditions upon cessation of the CO_2_ delivery. Our results are consistent with previous MRI studies that measured CVR in response to hypercapnia in healthy participants (Mark et al., 2010; De Vis et al., 2015; Zhou et al., 2015; Aslan et al., 2010; Bulte et al., 2012; Gauthier et al., 2013). However, the majority of these previous CVR studies use blood oxygenation level-dependent (BOLD) imaging or inhalation of fixed CO_2_, whereas the current study uses a pCASL protocol with prospective end-tidal targeting system which allows for precise and repeatable manipulation of P_ET_CO_2_. Our study administered hypercapnia for 3 min, and only the last 2 minutes of hypercapnia were used to calculate CVR as it provided a more stable measurement for CBF to reach a steady state. As P_ET_CO_2_ is a surrogate for the partial pressure of CO_2_ in the blood, including the time before steady-state when CO_2_ in the blood has yet to be saturated to affect the blood vessels would hinder the CVR results (Burley et al., 2020). Therefore, we recommend using the last 2-min label/control pairs during hypercapnia when performing CVR analysis. Unexpectedly, not all of our participants experienced an increase in CBF during hypercapnia. Most notable was the hippocampus in which four participants experienced reduced hippocampal CBF during CVR. We suspect the hippocampal regions experienced inappropriate vasoconstriction during hypercapnia due to stress response and/or inability to open adequate potassium channels that reduce intracellular calcium via hyperpolarization (Yoon et al., 2000). Further, brain parenchymal volume has been documented to increase in response to hypercapnia (van der Kleij et al., 2020), yet it is unclear if these effects are global or region-specific to the brain.

### Limitations

The order of pCASL protocols was not counter-balanced in this study because CVR testing required participants to exit the scanner and be fitted for a CO_2_ gas delivery mask. With this limitation, pCASL-HCP was always performed before pCASL-MOD. Further, participants were instructed to breathe deeply only during pCASL-MOD with the CVR testing CO_2_ gas delivery a mask to ensure accurate targeting of P_ET_CO_2_. This could have induced a respiratory-autonomic-cardiovascular response, which aside from differences in PLD, could add insight into the explanation of lower CBF measurements from our pCASL-MOD protocol. Lastly, this study was limited to midlife adults, and future studies should investigate the agreement in resting CBF between pCASL-HCP and pCASL-MOD in different age groups, non-healthy populations, and during interventions known to alter CBF. We expect similar resting CBF findings from the pCASL-MOD protocol in older adults (≥65 years old) but improved accuracy in younger adults (<45 years old) as this age group is less affected by differences in PLD (Hu et al., 2020). While the expected reliability of pCASL-MOD on measuring resting CBF in those with pre-existing diseases or undergoing an intervention that may improve CBF is less predictable, we do reason that there is an added benefit of measuring CVR as it can be more sensitive than CBF in predicting future cognitive decline (Kim et al., 2021).

## Conclusion

The utility of MRI for brain health, particularly the ability of pCASL to measure CBF at rest and in response to vasodilatory stimuli such as CVR, is an emerging area of research interest. Our study sought to advance the field by introducing our pCASL-MOD protocol to measure CBF during hypercapnia-induced CVR testing in midlife adults. Our pCASL-MOD protocol reliably measured resting GM CBF, WM CBF, and hippocampal CBF, and is suitable to measure the relative change in CBF from rest to hypercapnia during CVR testing as a primary outcome.

## Data Availability

The raw data supporting the conclusions of this article will be made available by the authors, without undue reservation.

## References

[B1] Alexopoulos P. Sorg C. Förschler A. Grimmer T. Skokou M. Wohlschläger A. (2012). Perfusion abnormalities in mild cognitive impairment and mild dementia in Alzheimer’s disease measured by pulsed arterial spin labeling MRI. Eur. Arch. Psychiatry Clin. Neurosci. 262, 69–77. 10.1007/s00406-011-0226-2 21786091

[B2] Alsop D. C. Dai W. Grossman M. Detre J. A. (2010). Arterial spin labeling blood flow MRI: its role in the early characterization of Alzheimer’s disease. J. Alzheimers Dis. 20, 871–880. 10.3233/JAD-2010-091699 20413865 PMC3643892

[B3] Alsop D. C. Detre J. A. Golay X. Günther M. Hendrikse J. Hernandez-Garcia L. (2015). Recommended implementation of arterial spin labeled perfusion MRI for clinical applications: a consensus of the ISMRM perfusion study group and the European consortium for ASL in dementia. Magn. Reson Med. 73, 102–116. 10.1002/mrm.25197 24715426 PMC4190138

[B4] Aslan S. Xu F. Wang P. L. Uh J. Yezhuvath U. S. van Osch M. (2010). Estimation of labeling efficiency in pseudocontinuous arterial spin labeling. Magnetic Reson. Med. 63, 765–771. 10.1002/mrm.22245 PMC2922009 20187183

[B5] Asllani I. Habeck C. Scarmeas N. Borogovac A. Brown T. R. Stern Y. (2008). Multivariate and univariate analysis of continuous arterial spin labeling perfusion MRI in Alzheimer’s disease. J. Cereb. Blood Flow. Metab. 28, 725–736. 10.1038/sj.jcbfm.9600570 17960142 PMC2711077

[B6] Bladt P. van Osch M. J. P. Clement P. Achten E. Sijbers J. den Dekker A. J. (2020). Supporting measurements or more averages? How to quantify cerebral blood flow most reliably in 5 minutes by arterial spin labeling. Magn. Reson Med. 84, 2523–2536. 10.1002/mrm.28314 32424947 PMC7402018

[B7] Bookheimer S. Y. Salat D. H. Terpstra M. Ances B. M. Barch D. M. Buckner R. L. (2019). The lifespan human connectome Project in aging: an overview. Neuroimage 185, 335–348. 10.1016/j.neuroimage.2018.10.009 30332613 PMC6649668

[B8] Bulte D. P. Kelly M. Germuska M. Xie J. Chappell M. A. Okell T. W. (2012). Quantitative measurement of cerebral physiology using respiratory-calibrated MRI. NeuroImage 60, 582–591. 10.1016/j.neuroimage.2011.12.017 22209811 PMC7100043

[B9] Burley C. V. Lucas R. A. I. Whittaker A. C. Mullinger K. Lucas S. J. E. (2020). The CO2 stimulus duration and steady-state time point used for data extraction alters the cerebrovascular reactivity outcome measure. Exp. Physiol. 105, 893–903. 10.1113/EP087883 32083357

[B10] Calamante F. Thomas D. L. Pell G. S. Wiersma J. Turner R. (1999). Measuring cerebral blood flow using magnetic resonance imaging techniques. J. Cereb. Blood Flow. Metab. 19, 701–735. 10.1097/00004647-199907000-00001 10413026

[B11] Chappell M. A. Groves A. R. Whitcher B. Woolrich M. (2009). Variational bayesian inference for a nonlinear forward model. IEEE Trans. Signal Process. 57, 223–236. 10.1109/tsp.2008.2005752

[B12] Chen Y. Wang D. J. J. Detre J. A. (2011). Test-retest reliability of arterial spin labeling with common labeling strategies. J. Magn. Reson Imaging 33, 940–949. 10.1002/jmri.22345 21448961 PMC3069716

[B13] Clement P. Mutsaerts H.-J. Václavů L. Ghariq E. Pizzini F. B. Smits M. (2018). Variability of physiological brain perfusion in healthy subjects – a systematic review of modifiers. Considerations for multi-center ASL studies. J. Cereb. Blood Flow. Metab. 38, 1418–1437. 10.1177/0271678X17702156 28393659 PMC6120130

[B14] Clement P. Petr J. Dijsselhof M. B. J. Padrela B. Pasternak M. Dolui S. (2022). A beginner’s guide to arterial spin labeling (ASL) image processing. Front. Radiology 2, 929533. 10.3389/fradi.2022.929533 PMC10365107 37492666

[B15] Dai W. Garcia D. de Bazelaire C. Alsop D. C. (2008). Continuous flow-driven inversion for arterial spin labeling using pulsed radio frequency and gradient fields. Magnetic Reson. Med. 60, 1488–1497. 10.1002/mrm.21790 PMC2750002 19025913

[B16] De Vis J. b. Hendrikse J. Bhogal A. Adams A. Kappelle L. J. Petersen E. T. (2015). Age-related changes in brain hemodynamics; A calibrated MRI study. Hum. Brain Mapp. 36, 3973–3987. 10.1002/hbm.22891 26177724 PMC6869092

[B17] Donahue M. J. Lu H. Jones C. K. Pekar J. J. van Zijl P. C. M. (2006). An account of the discrepancy between MRI and PET cerebral blood flow measures. A high-field MRI investigation. NMR Biomed. 19, 1043–1054. 10.1002/nbm.1075 16948114

[B18] Fierstra J. Sobczyk O. Battisti-Charbonney A. Mandell D. M. Poublanc J. Crawley A. P. (2013). Measuring cerebrovascular reactivity: what stimulus to use? J. Physiol. 591, 5809–5821. 10.1113/jphysiol.2013.259150 24081155 PMC3872753

[B19] Filley C. (2012). The behavioral neurology of white matter. USA: Oxford University Press.

[B20] Fisher J. A. (2016). The CO2 stimulus for cerebrovascular reactivity: fixing inspired concentrations vs. targeting end-tidal partial pressures. J. Cereb. Blood Flow. Metab. 36, 1004–1011. 10.1177/0271678X16639326 27000209 PMC4908627

[B21] Fisher J. A. Mikulis D. J. (2021). Cerebrovascular reactivity: purpose, optimizing methods, and limitations to interpretation – a personal 20-year odyssey of (Re)searching. Front. Physiology 12, 629651. 10.3389/fphys.2021.629651 PMC8047146 33868001

[B22] Fjell A. M. McEvoy L. Holland D. Dale A. M. Walhovd K. B. Alzheimer's Disease Neuroimaging Initiative (2014). What is normal in normal aging? Effects of aging, amyloid and Alzheimer’s disease on the cerebral cortex and the Hippocampus. Prog. Neurobiol. 117, 20–40. 10.1016/j.pneurobio.2014.02.004 24548606 PMC4343307

[B23] Gauthier C. J. Madjar C. Desjardins-Crépeau L. Bellec P. Bherer L. Hoge R. D. (2013). Age dependence of hemodynamic response characteristics in human functional magnetic resonance imaging. Neurobiol. Aging 34, 1469–1485. 10.1016/j.neurobiolaging.2012.11.002 23218565

[B24] Glasser M. F. Smith S. M. Marcus D. S. Andersson J. L. R. Auerbach E. J. Behrens T. E. J. (2016). The Human Connectome Project’s neuroimaging approach. Nat. Neurosci. 19, 1175–1187. 10.1038/nn.4361 27571196 PMC6172654

[B25] Haller S. Zaharchuk G. Thomas D. L. Lovblad K. O. Barkhof F. Golay X. (2016). Arterial spin labeling perfusion of the brain: emerging clinical applications. Radiology 281, 337–356. 10.1148/radiol.2016150789 27755938

[B26] Harms M. P. Somerville L. H. Ances B. M. Andersson J. Barch D. M. Bastiani M. (2018). Extending the human connectome Project across ages: imaging protocols for the lifespan development and aging projects. Neuroimage 183, 972–984. 10.1016/j.neuroimage.2018.09.060 30261308 PMC6484842

[B27] Heijtel D. F. R. Mutsaerts HJMM Bakker E. Schober P. Stevens M. F. Petersen E. T. (2014). Accuracy and precision of pseudo-continuous arterial spin labeling perfusion during baseline and hypercapnia: a head-to-head comparison with ¹⁵O H₂O positron emission tomography. NeuroImage 92, 182–192. 10.1016/j.neuroimage.2014.02.011 24531046

[B28] Hu Y. Lv F. Li Q. Liu R. (2020). Effect of post-labeling delay on regional cerebral blood flow in arterial spin-labeling MR imaging. Med. Baltim. 99, e20463. 10.1097/MD.0000000000020463 PMC7337483 32629629

[B29] Iadecola C. (2004). Neurovascular regulation in the normal brain and in Alzheimer’s disease. Nat. Rev. Neurosci. 5, 347–360. 10.1038/nrn1387 15100718

[B30] Juttukonda M. R. Li B. Almaktoum R. Stephens K. A. Yochim K. M. Yacoub E. (2021). Characterizing cerebral hemodynamics across the adult lifespan with arterial spin labeling MRI data from the Human Connectome Project-Aging. Neuroimage 230, 117807. 10.1016/j.neuroimage.2021.117807 33524575 PMC8185881

[B31] Kamano H. Yoshiura T. Hiwatashi A. Abe K. Togao O. Yamashita K. (2013). Arterial spin labeling in patients with chronic cerebral artery steno-occlusive disease: correlation with (15)O-PET. Acta Radiol. 54, 99–106. 10.1258/ar.2012.120450 23091237

[B32] Kim D. Hughes T. M. Lipford M. E. Craft S. Baker L. D. Lockhart S. N. (2021). Relationship between cerebrovascular reactivity and cognition among people with risk of cognitive decline. Front. Physiol. 12, 645342. 10.3389/fphys.2021.645342 34135768 PMC8201407

[B33] Kochanowicz J. Mariak Z. Rutkowski R. Turek G. Lysoń T. Krejza J. (2009). Age and sex dependency of blood flow velocity in the internal carotid artery. Neurol. Neurochir. Pol. 43, 3–8. 10.1134/S0362119714050041 19353438

[B34] Li X. Wang D. Auerbach E. J. Moeller S. Ugurbil K. Metzger G. J. (2015). Theoretical and experimental evaluation of multi-band EPI for high-resolution whole brain pCASL imaging. Neuroimage 106, 170–181. 10.1016/j.neuroimage.2014.10.029 25462690 PMC4337884

[B35] Mark C. I. Slessarev M. Ito S. Han J. Fisher J. A. Pike G. B. (2010). Precise control of end-tidal carbon dioxide and oxygen improves BOLD and ASL cerebrovascular reactivity measures. Magn. Reson Med. 64, 749–756. 10.1002/mrm.22405 20648687

[B36] Melzer T. R. Watts R. MacAskill M. R. Pearson J. F. Rüeger S. Pitcher T. L. (2011). Arterial spin labelling reveals an abnormal cerebral perfusion pattern in Parkinson’s disease. Brain 134, 845–855. 10.1093/brain/awq377 21310726 PMC3105489

[B37] Mezue M. Segerdahl A. R. Okell T. W. Chappell M. A. Kelly M. E. Tracey I. (2014). Optimization and reliability of multiple postlabeling delay pseudo-continuous arterial spin labeling during rest and stimulus-induced functional task activation. J. Cereb. Blood Flow. Metab. 34, 1919–1927. 10.1038/jcbfm.2014.163 25269517 PMC4269746

[B38] Portegies M. L. P. de Bruijn RFAG Hofman A. Koudstaal P. J. Ikram M. A. (2014). Cerebral vasomotor reactivity and risk of mortality: the Rotterdam Study. Stroke 45, 42–47. 10.1161/STROKEAHA.113.002348 24203842

[B39] Ruitenberg A. den Heijer T. Bakker S. L. M. van Swieten J. C. Koudstaal P. J. Hofman A. (2005). Cerebral hypoperfusion and clinical onset of dementia: the Rotterdam Study. Ann. Neurol. 57, 789–794. 10.1002/ana.20493 15929050

[B40] Scheef L. Manka C. Daamen M. Kühn K. U. Maier W. Schild H. H. (2010). Resting-state perfusion in nonmedicated schizophrenic patients: a continuous arterial spin-labeling 3.0-T MR study. Radiology 256, 253–260. 10.1148/radiol.10091224 20505069

[B41] Somerville L. H. Bookheimer S. Y. Buckner R. L. Burgess G. C. Curtiss S. W. Dapretto M. (2018). The Lifespan Human Connectome Project in Development: a large-scale study of brain connectivity development in 5-21 year olds. Neuroimage 183, 456–468. 10.1016/j.neuroimage.2018.08.050 30142446 PMC6416053

[B42] Tosun D. Mojabi P. Weiner M. W. Schuff N. (2010). Joint analysis of structural and perfusion MRI for cognitive assessment and classification of Alzheimer’s disease and normal aging. Neuroimage 52, 186–197. 10.1016/j.neuroimage.2010.04.033 20406691 PMC4667806

[B43] Uğurbil K. Xu J. Auerbach E. J. Moeller S. Vu A. T. Duarte-Carvajalino J. M. (2013). Pushing spatial and temporal resolution for functional and diffusion MRI in the Human Connectome Project. NeuroImage 80, 80–104. 10.1016/j.neuroimage.2013.05.012 23702417 PMC3740184

[B44] van der Kleij L. A. De Vis J. B. de Bresser J. Hendrikse J. Siero J. C. W. (2020). Arterial CO2 pressure changes during hypercapnia are associated with changes in brain parenchymal volume. Eur. Radiol. Exp. 4, 17. 10.1186/s41747-020-0144-z 32147754 PMC7061094

[B45] Van Essen D. C. Smith S. M. Barch D. M. Behrens T. E. J. Yacoub E. Ugurbil K. (2013). The Wu-minn human connectome Project: an overview. NeuroImage 80, 62–79. 10.1016/j.neuroimage.2013.05.041 23684880 PMC3724347

[B46] Van Essen D. C. Ugurbil K. Auerbach E. Barch D. Behrens T. E. J. Bucholz R. (2012). The Human Connectome Project: a data acquisition perspective. NeuroImage 62, 2222–2231. 10.1016/j.neuroimage.2012.02.018 22366334 PMC3606888

[B47] Wierenga C. E. Hays C. C. Zlatar Z. Z. (2014). Cerebral blood flow measured by arterial spin labeling MRI as a preclinical marker of Alzheimer’s disease. J. Alzheimers Dis. 42, S411–S419. 10.3233/JAD-141467 25159672 PMC5279221

[B48] Winter J. D. Fierstra J. Dorner S. Fisher J. A. St Lawrence K. S. Kassner A. (2010). Feasibility and precision of cerebral blood flow and cerebrovascular reactivity MRI measurements using a computer-controlled gas delivery system in an anesthetised juvenile animal model. J. Magn. Reson Imaging 32, 1068–1075. 10.1002/jmri.22230 21031510

[B49] Wolters F. J. Zonneveld H. I. Hofman A. van der Lugt A. Koudstaal P. J. Vernooij M. W. (2017). Cerebral perfusion and the risk of dementia: a population-based study. Circulation 136, 719–728. 10.1161/CIRCULATIONAHA.117.027448 28588075

[B50] Wu B. Lou X. Wu X. Ma L. (2014). Intra- and interscanner reliability and reproducibility of 3D whole-brain pseudo-continuous arterial spin-labeling MR perfusion at 3T. J. Magn. Reson Imaging 39, 402–409. 10.1002/jmri.24175 23723043

[B51] Wu W.-C. Fernández-Seara M. Detre J. A. Wehrli F. W. Wang J. (2007). A theoretical and experimental investigation of the tagging efficiency of pseudocontinuous arterial spin labeling. Magn. Reson Med. 58, 1020–1027. 10.1002/mrm.21403 17969096

[B52] Xu G. Rowley H. A. Wu G. Alsop D. C. Shankaranarayanan A. Dowling M. (2010). Reliability and precision of pseudo-continuous arterial spin labeling perfusion MRI on 3.0 T and comparison with 15O-water PET in elderly subjects at risk for Alzheimer’s disease. NMR Biomed. 23, 286–293. 10.1002/nbm.1462 19953503 PMC2843795

[B53] Yoon S. Zuccarello M. Rapoport R. M. (2000). Reversal of hypercapnia induces KATP channel and NO-independent constriction of basilar artery in rabbits with acute metabolic alkalosis. General Pharmacol. Vasc. Syst. 35, 325–332. 10.1016/s0306-3623(02)00111-8 11922963

[B54] Zhou Y. Rodgers Z. B. Kuo A. H. (2015). Cerebrovascular reactivity measured with arterial spin labeling and blood oxygen level dependent techniques. Magn. Reson Imaging 33, 566–576. 10.1016/j.mri.2015.02.018 25708263 PMC4426232

